# Antibacterial and Antioxidant Activities of Ursolic Acid and Derivatives

**DOI:** 10.3390/molecules19011317

**Published:** 2014-01-21

**Authors:** Patrícia G.G. do Nascimento, Telma L.G. Lemos, Ayla M.C. Bizerra, Ângela M.C. Arriaga, Daniele A. Ferreira, Gilvandete M.P. Santiago, Raimundo Braz-Filho, José Galberto M. Costa

**Affiliations:** 1 Departamento de Química Orgânica e Inorgânica, Universidade Federal do Ceará, Fortaleza-CE 60451-970, Brazil; E-Mails: georgina.quimica@gmail.com (P.G.G.N.); aylamarcia@yahoo.com.br (A.M.C.B.); angelamcarriaga@yahoo.com.br (A.M.C.A.); dafufc@yahoo.com.br (D.A.F.); 2 Departamento de Farmácia, Universidade Federal do Ceará, Fortaleza-CE 60430-370, Brazil; E-Mail: gil@ufc.br; 3 Laboratório de Ciências Químicas (LCQUI)-CCT-UENF/PPGQO-DEQUIM-UFRRJ, Campos dos Goytacazes-RJ 28013-602, Brazil; E-Mail: braz@uenf.br; 4 Laboratório de Pesquisa de Produtos Naturais, Universidade Regional do Cariri, Crato-CE 63105-000, Brazil; E-Mail: galberto.martins@gmail.com

**Keywords:** *Sambucus australis*, ursolic acid, antioxidant activity, antibacterial activity

## Abstract

Ursolic acid, an important bioactive compound, was isolated from ethanol extract of aerial parts of *Sambucus australis*. In order to develop bioactive ursolic acid derivatives, two semi-synthetic compounds were obtained through modification at C-3. The antibacterial activity of the ursolic acid and its derivatives was investigated. The microdilution method was used for determination of the minimal inhibitory concentration (MIC), against twelve bacterial strains. The influence of ursolic acid and its derivatives on the susceptibility of some bacterial pathogens to the aminoglycosides antibiotics neomycin, amikacin, kanamycin and gentamicin was evaluated. The most representative synergistic effect was observed by 3β-formyloxy-urs-12-en-28-oic acid at the concentration of 64 μg/mL in combination with kanamycin against *Escherichia coli* (27), a multidrug-resistant clinical isolate from sputum, with reduction of MIC value from 128 μg/mL to 8 μg/mL. Ursolic acid and its derivatives were examined for their radical scavenger activity using the DPPH assay, and showed significant activity.

## 1. Introduction

Ursolic acid (**1**), an ursane-type pentacyclic triterpene, is a constituent of certain medicinal herbs and is also found in fruits [1]. This triterpenoid is the major secondary metabolite isolated from the ethanol extract of aerial parts of *Sambucus australis* Cham. & Schltdl (Caprifoliaceae), a shrub, popularly known as “sabugueiro”.

It is well known to possess a wide range of biological activities including anti-inflammatory [2], anticancer [3], hypoglycemic [4], antiprotozoal against *Plasmodium falciparum* [5], antioxidant [1,6,7], antibacterial [8], and prevents abdominal adiposity [9]. The antibacterial properties of pentacyclic triterpenes and their derivatives have been extensively studied [10,11,12,13], and the activity of these compounds also resides in their potential to enhance bacterial susceptibility to other compounds, including antibiotics [14].

Antibiotic resistance is a serious problem in the area of public health. The search for new therapeutic agents that can help patients infected by bacterial agents is a challenge for all professionals in the field. The use of natural products is an alternative that can produce good results.

The production of oxygen and free radicals in the body, probably involves the development of many diseases such as inflammation, cancer, rheumatoid arthritis, Parkinson's and Alzheimer's diseases [15]. The natural antioxidants cause less toxic side effect and can provide protection against oxidative degradation by decrease free radicals in cosmetic, pharmaceutical, and food.

The aim of the present study was to prepare derivatives of ursolic acid (**1**), and to investigate the antibacterial and scavenger activities using DPPH assay of one **1** and its derivatives **1a** and **1b**. The influence of the compounds **1**, **1a** and **1b** on the susceptibility of several Gram-positive and Gram-negative bacteria towards the aminoglycoside antibiotics neomycin, amikacin, kanamycin and gentamicin was also evaluated.

## 2. Results and Discussion

### 2.1. Synthesis

**Scheme 1:**
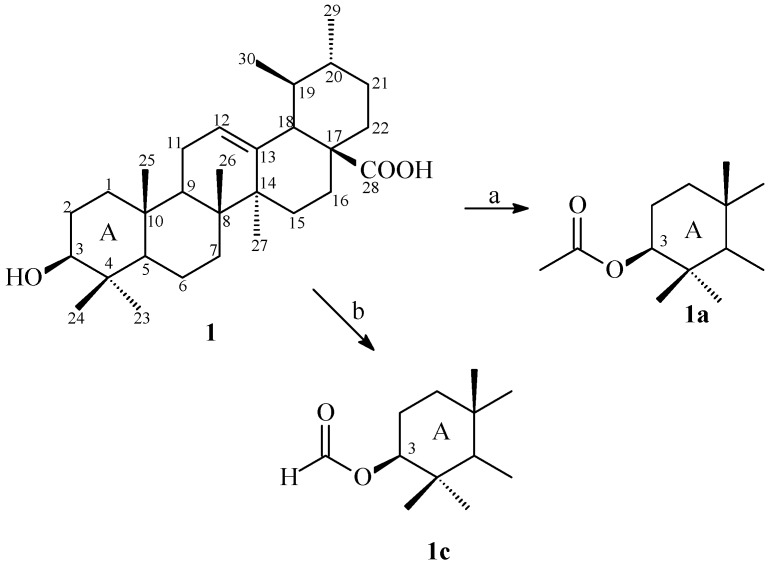
Synthesis of ursolic acid derivatives.

Ethanol extract of the aerial parts of *S. australis* (SAEtOH) was fractionated and purified by classical chromatographic methods to isolate the triterpenoid ursolic acid (**1**) [16]. In order to develop bioactive ursolic acid derivatives, two semi-synthetic compounds were obtained through modification at C-3. The structural changes involving the hydroxyl group were acetylation and formylation (Scheme 1). The structures of all compounds were established by 1D ^1^H and ^13^C (and DEPT) and 2D HSQC and HMBC NMR spectral data and by comparing their spectroscopy data with the literature [16,17,18]. 

The treatment of (**1**) with acetic anhydride and pyridine at room temperature afforded 3β-acetoxy-urs-12-en-28-oic acid (**1a**) in quantitative yield. The ^1^H-NMR spectrum of **1a** showed a signal at δ_H_ 2.05 typical of hydrogen of acetyl group. The presence of this group was further confirmed by the appearance of the signal at δ_C_ 170.9 in the ^13^C- NMR spectrum [17]. Analysis of the ^1^H-NMR spectrum of 3β-formiloxy-urs-12-en-28-oic acid (**1b**) showed a signal at δ_H_ 8.12 for the formyl group confirmed by the presence of a signal at δ_C_ 161.3 attributed to a carbonyl [18].

*Reagents and Conditions*: (a) Ac_2_O, pyridine/rt/24 h; (b) HCO_2_H, perchloric acid/60 °C/4 h.

### 2.2. Antimicrobial Activity and Evaluation of the Modulatory Activity by Direct Contact

Ursolic acid (**1**), 3β-acetoxy-urs-12-en-28-oic acid (**1a**) and 3β-formyloxy-urs-12-en-28-oic acid (**1b**) were tested for antibacterial activity against twelve bacterial strains (different strains of *Staphylococcus aureus*, *Bacillus cereus*, two strains of *Escherichia coli*, *Pseudomonas aeruginosa*, *Aeromonas caveae*, *Klebsiella pneumoniae*, *Shigella flexneri*, *Vibrio colareae* and *Listeria monocytogenes*), by employing the microdilution method. The corresponding minimum inhibitory concentration (MIC) values of (**1**), (**1a**) and (**1b**) are shown in Table 1.

Although the antibacterial activity of ursolic acid (**1**) against several bacterial strains has been reported in the literature [8,10,11,12], in our case compound **1** showed activity against six bacterial strains, and the best result was found against *S. aureus* (ATCC 6538), with a MIC value of 32 μg/mL. Ursolic acid (**1**) was also effective against *E. coli* (ATCC 25922), *K. pneumoniae* and *S. flexneri* with a MIC value of 64 μg/mL in the three cases.

3β-Acetoxy-urs-12-en-28-oic acid (**1a**) showed significant activity (in terms of clinical MIC ≤ 1,024 μg/mL) against all tested strains except for *S. aureus* (ATCC 12624). The best results were found against *E. coli* (ATCC 25922) and *S. flexneri* with a MIC value of 32 μg/mL in both cases, *K. pneumoniae* and *S. aureus* (ATCC 6538) with MIC values of 64 μg/mL and 128 μg/mL, respectively (Table 1). The literature reports activity against *Streptococcus pneumoniae* (ATCC 6305) for compound (**1a**) [11], however, this is the first report on the antibacterial activity of the derivative **1a** on the bacterial strains used.

In the present study, 3β-formyloxy-urs-12-en-28-oic acid (**1b**) presented significant activity from a clinical point of view against ten evaluated bacterial strains, with emphasis against *S. aureus* (ATCC 6538), *A. caveae* and *L. monocytogenes* with MIC values of 256 μg/mL, and *K pneumoniae* and *S. flexneri* with MIC values of 128 μg/mL (Table 1). 

**Table 1:** Values of the minimal inhibitory concentration (MIC) of compounds **1**, **1a** and **1b**.

Bacterial strains	MIC (µg/mL)		
	1	1a	1b
*Staphylococcus aureus* (ATCC 12692)	≥1024	256	512
*Staphylococcus aureus* (ATCC 12624)	≥1024	≥1024	≥1024
*Staphylococcus aureus* (ATCC 6538)	32	128	256
*Bacillus cereus* (ATCC 33018)	≥1024	512	512
*Escherichia coli* (ATCC 25922)	64	32	512
*Escherichia coli* (ATCC 27)	512	256	512
*Pseudomonas aeruginosa* (ATCC 15442)	512	512	512
*Aeromonas caveae* (ATCC 15468)	≥1024	256	256
*Klebsiella pneumoniae* (ATCC 10031)	64	64	128
*Shigella flexneri* (ATCC 12022)	64	32	128
*Vibrio colareae* (ATCC 15748)	≥1024	512	≥1024
*Listeria monocytogenes* (ATCC 19117)	≥1024	256	256

(**1**) ursolic acid, (**1a**) 3β-acetoxy-urs-12-en-28-oic acid, (**1b**) 3β-formyloxy-urs-12-en-28-oic acid.

Interestingly, among all evaluated bacterial strains in this study, *S. aureus* (ATCC 12624) was the most resistant to the tested compounds **1**, **1a** and **1b**.

The effects on the MIC values of the association of compounds **1**, **1a** and **1b** with aminoglycoside antibiotics are shown in Table 2. In these assays the compounds that showed MIC ≤ 1024 μg/mL in the MIC determination were used.

Table 2 indicate a synergism between ursolic acid (**1**) and the aminoglycoside antibiotics in almost all microorganisms, except for gentamicin and amikacin against *E. coli* (ATCC 25922), and kanamycin against *P. aeruginosa* and *K. pneumoniae*. Ursolic acid (**1**) was found to enhance the susceptibility of *Staphylococcus aureus*, *Staphylococcus epidermidis* and *Listeria monocytogenes* to the β-lactam antibiotics ampicilin and oxacilin [14], but there is no report in the literature about this compound acting synergistically with the aminoglycoside antibiotics neomycin, amikacin, kanamycin and gentamicin.

The association of 3β-acetoxy-urs-12-en-28-oic acid (**1a**) with the aminoglycoside antibiotics increased the effect of these antibiotics against the strains evaluated; thus **1a** may be considered an excellent enhancer of the mechanism of action of these aminoglycoside antibiotics.

The association of 3β-formyloxy-urs-12-en-28-oic acid (**1b**) with the aminoglycoside antibiotics showed synergistic effects against several microorganisms. It is also possible to demonstrate that there was no potential interference on the activity of gentamicin against *B. cereus* and *S. aureus* (ATCC 12692) when associated to 3β-formyloxy-urs-12-en-28-oic acid (**1b**) and the associations with amikacin against *K. pneumoniae* and kanamycin against *L. monocytogenes*.

**Table 2:** Combinating testing of ursolic acid (**1**), 3β-acetoxy-urs-12-en-28-oic acid (**1a**) and 3β-formiloxy-urs-12-en-28-oic acid (**1b**) plus neomycin, amikacin, kanamycin and gentamicin against bacterial strains.

Bacterial strains	Combination tested Antibiotic + Substance (µg/mL)	MIC (µg/mL)			
		Neomycin	Amikacin	Kanamycin	Gentamicin
*S. aureus* 12692	*	128	64	64	128
	**1a** (32)	16	4	8	32
	**1b** (64)	64	32	16	128
*S. aureus* 6538	*	128	128	64	64
	**1** (4)	18	32	16	4
	**1a** (16)	16	32	16	16
	**1b** (32)	32	64	32	16
*B. cereus* 33018	*	128	128	64	64
	**1a** (64)	64	32	8	16
	**1b** (64)	64	64	32	64
*E. coli* 25922	*	128	64	128	64
	**1** (8)	32	64	64	64
	**1a** (4)	32	32	16	16
	**1b** (64)	64	32	64	32
*E. coli* 27	*	64	128	128	64
	**1** (8)	4	32	16	32
	**1a** (32)	16	32	32	8
	**1b** (64)	32	16	8	8
*P. aeruginosa* 15442	*	128	128	64	32
	**1** (64)	64	32	64	16
	**1a** (64)	64	32	16	4
	**1b **(64)	32	32	16	8
*A. caveae* 15468	*	64	128	128	128
	**1a** (32)	8	16	32	32
	**1b** (64)	16	64	32	32
*K. pneumonia* 10031	*	256	64	64	128
	**1** (8)	128	32	64	16
	**1a** (8)	64	32	16	64
	**1b** (16)	128	64	32	64
*S. flexneri* 12022	*	512	128	64	64
	**1** (8)	64	32	16	16
	**1a** (4)	256	64	16	8
	**1b** (16)	128	32	16	32
*L. monocytogenes* 19117	*	128	128	64	64
	**1a** (32)	16	32	32	8
	**1b** (32)	32	64	64	16
*V. colareae* 15748	*	64	32	128	128
	**1a** (64)	16	4	16	32

* only antibiotics.

### 2.3. Antioxidant Activity

Compounds **1** and **1a** showed antioxidant activity by inhibiting DPPH. The compounds strongly scavenged DPPH radical, with IC_50_ values of 5.97 × 10^−2^ ± 1 × 10^−3^ and 0.73 ± 9.3 × 10^−2^ mg/mL, respectively. Trolox and Vitamin C, used as positive controls, showed IC_50_ values of 2.6 × 10^−3^ ± 2.3 × 10^−4^ and 4.3 × 10^−2^ ± 1.9 × 10^−2^ mg/mL, respectively (Table 3). Compound **1b** was inactive in the DPPH assay.

**Table 3:** IC_50_ values of ursolic acid (**1)** and derivatives (**1a** and **1b**).

Samples	IC_50_ (mg/mL)
**1**	5.97 × 10^−2^ ± 1 × 10^−3^
**1a**	0.73 ± 9.3 × 10^−2^
**1b**	not active
Trolox	2.6 × 10^−3^ ± 2.3 × 10^−4^
Vitamin C	4.3 × 10^−2^ ± 1.9 × 10^−2^

## 3. Experimental

### 3.1. General

Melting points were determined on a digital Mettler Toledo FP82HT apparatus and are uncorrected. The IR spectra were measured in KBr pellets using a Perkin-Elmer FT-IR Spectrum 1000. A Bruker^®^ Avance DRX 500 and a Bruker^®^ Avance DPX 300 spectrometers, operating at 500 MHz and 300 MHz for ^1^H-NMR, and 125 MHz and 75 MHz for ^13^C-NMR were used for experiments 1D and 2D with chemical shifts given in ppm. The spectra were run using CDCl_3_ and pyridine-*d_5_* as the solvent. Chemical shifts, measured on the δ scale. The absorptions in the region of the ultraviolet (UV) visible were obtained on a Varian Cary 50 Conc spectrophotometer. The low resolution mass spectra were obtained by electron impact at 70 eV spectrometer with Shimadzu a QP 5000, DI-50 instrument. Silica gel 60 (70–230 mesh) was used for column chromatography, and thin layer chromatography (TLC) was performed on precoated silica gel G60 F_254_ by detection by spraying with vanillin in perchloric acid/ethanol. All solvents used for chromatography were from Synth. The microbiological culture media were purchased from Fundação Oswaldo Cruz—FIOCRUZ (Rio de Janeiro, Brazil).

### 3.2. Plant Material

The aerial parts of *Sambucus australis* were collected in Guaramiranga County, State of Ceará, northeast Brazil. A voucher specimen (#EAC15002) has been deposited at the Herbarium Prisco Bezerra, Department of Biology, Federal University of Ceará, Brazil.

### 3.3. Extraction and Isolation

Aerial parts including leaves and flowers of *Sambucus australis* were extracted with ethyl acetate (1,000 g/1,500 mL) for 72 h at 25 °C, followed by ethanol (1,000 g/5,000 mL) for 72 h at 25 °C to yield: SAEtOAc, (3.9 g, 0.39%) and SAEtOH, (1.0 g, 0.1%), respectively. The ethanol extract (SAEtOH) (1.0 g) was chromatographed on a silica gel column running a gradient of 100% ethyl acetate to 100% methanol. The fraction eluted with 100% ethyl acetate yield a solid material, which was further recrystallized in ethyl acetate to afforded ursolic acid (**1**, 180 mg, 18%); White solid; m.p. 279.0–281.1 °C; FTIR (cm^−1^): 3407, 2924, 2855, 1686, 1456, 1387. MS (EI): *m/z* (%) 456 (0.2), 248 (100), 203 (43), 133 (37), 44(83). ^13^C-NMR (C_5_D_5_N) δ ppm: 37.98 (C-1), 28.65 (C-2), 78.62 (C-3), 39.85) (C-4), 56.32 (C-5); 19.44 (C-6), 34.11 (C-7), 39.45 (C-8), 48.59 (C-9), 37.78 (C-10), 24.06 (C-11), 126.14 (C-12), 139.75 (C-13), 42.51 (C-14), 28.65 (C-15 ), 25.45 (C-16), 48.54 (C-17), 54.04 (C-18), 40.02 (C-19), 39.94 (C-20), 31.60 (C-21), 37.96 (C-22), 29.35 (C-23), 15.19 (C-24), 15.25 (C-25), 18.06 (C-26), 26.69 (C-27), 180.34 (C-28), 18.06 (C-29), 21.94 (C-30) in agreement with the literature [16].

### 3.4. Synthesis 3β-Acetoxy-urs-12-en-28-oic Acid (**1a**)

To a solution of ursolic acid (**1**, 50 mg, 0.109 mmol) in pyridine (0.5 mL) was added Ac_2_O (2 mL, 1.05 mmol). After stirring at room temperature for 24 h, the reaction mixture was quenched with saturated CuSO_4_ (20 mL) and extracted with EtOAc 3 × 30 mL. The combined extracts were washed with H_2_O and brine, dried over anhydrous Na_2_SO_4_, and evaporated under reduced pressure. The residue was purified by column chromatography on silica gel with *n*-hexane/ethyl acetate = 3:7 (v:v) to give a white solid; Yield 75% (40.0 mg); m.p. 175.3–178.1 °C. FTIR (cm^−1^): 3215, 2924, 1722, 1447, 1366; ^13^C-NMR (CDCl_3_) δ ppm: C-1 (36.94), C-2 (24.25), C-3 (81.17); C-4 (37.91); C-5 (55.51), C-6 (18.37), C-7 (33.24), C-8 (39.71), C-9 (47.68), C-10 (37.13), C-11 (23.50), C-12 (125.93), C-13 (138.18), C-14 (42.10), C-15 (28.20), C-16 (23.78), C-17 (48.18); C-18 (52.71), C-19 (39.23), C-20 (39.04), C-21 (30.81), C-22 (38.47), C-23 (28.29), C-24 (17.23), C-25 (15.60), C-26 (16.91), C-27 (23.80), C-28 (184.15), C-29 (17.33), C-30 (21.51); EIMS (*m/z*): 498 (0.1%, M^+^), 248 (17%), 43 (82%) in agreement with the literature [17].

### 3.5. Synthesis 3β-Formiloxy-urs-12-en-28-oic Acid (**1b**)

A solution of ursolic acid (**1**, 50 mg, 0.109 mmol) in 89.9% HCO_2_H (1.5 mL) and 70% perchloric acid (6 drops) was heated in an H_2_O bath at 60 °C for 4 h. The solution was removed from the bath and allowed to cool to about 40 °C. Ac_2_O was then added dropwise while the temperature was maintained between 55 and 60 °C until a large quantity of bubbles appeared (1 mL of Ac_2_O was required). The solution was then cooled to room temperature and poured into 10 ml of H_2_O, with stirring [19]. The precipitate was filtered under vacuum, washed with H_2_O, and dried to give a white solid; Yield 28% (23.1 mg); m.p. 117.3–119.4 °C; FTIR (cm^−1^): 2924, 1718, 1688, 1458, 1369; ^13^C-NMR (CDCl_3_) δ ppm: 38.53 (C-1), 23.55 (C-2), 81.35 (C-3), 37.92 (C-4), 55.57 (C-5), 18.46 (C-6), 33.11 (C-7), 39.83 (C-8), 47.75 (C-9), 37.18 (C-10), 23.97 (C-11), 125.94 (C-12), 138.27 (C-13), 42.25 (C-14), 29.94 (C-15), 24.33 (C-16), 48.22 (C-17), 52.84 (C-18), 39.10 (C-19), 39.29 (C-20), 30.86 (C-21), 36.96 (C-22), 28.27 (C-23), 21.40 (C-24), 15.75 (C-25), 16.94 (C-26), 23.84 (C-27), 183.18 (C-28), 17.25 (C-29), 17.30 (C-30) in agreement with the literature [18].

### 3.6. Antibacterial Activity and Minimal Inhibitory Concentration

The antibacterial activity of the ursolic acid and its derivatives were investigated employing a microdilution method, recommended by National Committee for Clinical and Laboratory Standards M7-A6 [20]. In tests were used eight standard strains of Gram (−) and four Gram (+), and two clinical isolates of multidrug-resistant *Escherichia coli* (27) (from sputum) and *Staphylococcus aureus* (6538) of the surgical wound. The brain heart infusion (BHI 3.8%) broth was used for the bacterial growth (24 h, 35 ± 2 °C). The inoculum was an overnight culture of each bacterial species in the BHI broth diluted in the same medium to a final concentration of approximately 1 × 10^8^ CFU/mL (0.5NTU – McFarland scale). After this, the suspension was dilluted to 1 × 10^6^ CFU/mL in 10% BHI. A total of 100 μL of each dilution was distributed in 96-well plates plus substance, achieving 5 × 10^5^ CFU/mL as the final concentration of the inoculums [21,22,23]. 

The initial solution of the ursolic acid and its derivatives were performed using 10 mg of each extract dissolved in 1 mL of dimethyl sulfoxide (DMSO) to obtain an initial concentration of 10 mg/mL. From this concentration, several dilutions were made in distilled water in order to obtain a stock solution of 1,024 μg/mL. Further serial dilutions were performed by the addition of the BHI broth to reach a final concentration in the range of 8–512 μg/mL). All experiments were performed in triplicate and the microdilution trays were incubated at 35 ± 2 °C for 24 h. The antibacterial activity was detected using a colorimetric method by adding 25 μL of the resauzurin staining (0.01%) aqueous solution in each well at the end of the incubation period [24]. The minimal inhibitory concentration (MIC) was defined as the lowest extract concentration able to inhibit the bacteria growth, as indicated by resauzurin staining (dead bacterial cells are not able to change the staining color by visual observation—blue to red).

### 3.7. Evaluation of the Modulatory Activity by Direct Contact

In order to evaluate of the ursolic acid and its derivatives as modulators of antibiotic resistance, the MICs values of aminoglycosides antibiotics neomycin, kanamycin, amikacin, and gentamicin against the analyzed strains were determined in the presence or absence of the extracts using the microdutiltion test. Subinhibitory concentrations (MIC 1/8) in 10% BHI were used. The antibiotic solutions (1024 μg/mL) were prepared in distillated water for use on the same day. A total of 100 μL of the antibiotic solution, using serial dilutions (1:2), was added to the wells containing 10% BHI and the diluted bacterial suspension (1:10). Microplates were incubated at 35 ± 2 °C for 24 h and the antibacterial activity was determined as described before [25]. 

### 3.8. Antioxidant Activity

The antioxidant activities of ursolic acid (**1**), and derivatives **1a** and **1b** were evaluated by measuring the reduction of the free radical 1,1-diphenyl-1-picrylhydrazyl (DPPH). The samples (1.0 to 1000.0 μg/mL) were dissolved in methanol and then added to a methanol solution of DPPH (60 mM) [26]. After 30 min, the UV absorbance of the resulting solutions was recorded at λ 517 nm. The experiment was performed in triplicate and the average absorption was noted for each concentration. Trolox and Vitamin C were used as the positive control. The free radical scavenging activity was calculated as a percentage inhibition of the DPPH radical by the sample or positive control. The IC_50_ value is the concentration required to scavenge 50% DPPH.

## 4. Conclusions

This study showed that ursolic acid (**1**) and some of its derivatives have significant antibacterial activity against several bacterial species, and that these compounds show synergistic activities with the aminoglycoside antibiotics neomycin, amikacin, kanamycin and gentamicin. These results suggest that *Sambucus australis* could be a potential natural source of free radical scavengers. 
